# Dropless After Cataract Surgery (DACS) for patients with difficulties using eye drops

**DOI:** 10.1038/s41433-024-03055-8

**Published:** 2024-04-12

**Authors:** Johannes Birtel, Guy Mole, Sher A. Aslam, Peter Charbel Issa

**Affiliations:** 1 Oxford Eye Hospital, Oxford University Hospitals NHS Foundation Trust, Oxford, UK; 2 Nuffield Laboratory of Ophthalmology, Nuffield Department of Clinical Neurosciences, University of Oxford, Oxford, UK; 3 Department of Ophthalmology, University Medical Center Hamburg-Eppendorf, Hamburg, Germany; 4 Department of Ophthalmology, Technical University Munich, Munich, Germany

**Keywords:** Lens diseases, Surgery

Eye drops are typically used in the postoperative management of patients undergoing cataract surgery. Even though this mode of administration may appear straightforward, it can present a challenge for patients to instil correctly [1]. While many surgeons choose to substitute topical antibiotics with an intracameral antibiotic solution at the end of surgery [2], control of postoperative inflammation is usually achieved via steroid and/or non-steroidal eye drops. This treatment burden may be avoided if patients receive a subconjunctival crystalline steroid depot [triamcinolone acetonide as 0.3 ml Adcortyl^®^ (10 mg/ml)] at the end of surgery [3]. We offered this treatment regimen to patients who would have required assistance for eye drop instillation, for reasons including arthritis, Parkinson’s disease, cognitive or intellectual disability, and who had no domestic support to aid correct application.

Data analysis was performed after 104 cataract surgeries in 81 patients (23 patients had surgery in both eyes) as a clinical audit at the Oxford Eye Hospital. Postoperative assesments one week, one, and three months after surgery included slit lamp examination and OCT imaging to document inflammation, intraocular pressure, presence of pseudophakic cystoid macular oedema and potential local side effects of the subconjunctival injection. Visual acuity was not deemed to be a relevant parameter in this cohort as assessment was often not reliable and/or because of the presence of ocular co-pathology. A total of 100 eyes were analysed after excluding four eyes: two patients did not attend follow up (one after immediate sequential bilateral cataract surgery) and one patient required additional surgery to remove a remaining nuclear fragment. Thirteen eyes received a higher steroid dose because more pronounced inflammation was expected (Table 1). All three postoperative appointments were attended after 73 surgeries and in the remaining, two of the scheduled assessments were attended.

**Table 1:** Deviation from standard subconjunctival (sc) injection of 3 ml Adcortyl (3 mg triamcinolone acetonide).

Deviation	Reason
Additional sc betamethasone	Intraoperative use of a Malyugin ring (*n* = 5) Intraoperative mild iris trauma (*n* = 2) Dense nucleus requiring high phaco energy (*n* = 2)
Higher Adcortyl dose	Intraoperative use of a Malyugin ring (5 mg; *n* = 1) Dense nucleus requiring high phaco energy (4 mg; *n* = 1) Black patient (4 mg; *n* = 1)
Higher Adcortyl dose + additional sc betamethasone	Previous Irvine–Gass syndrome in Black patient (5 mg; *n* = 1)

Minimal anterior chamber cells were observed one week after surgery, and no relevant inflammation was present at one and three months. No eye developed high intraocular pressure or showed local side effects from the subconjunctival injection, apart from a few with hyposphagma directly after the injection (not systematically documented). No patient required any additional anti-inflammatory treatment during the observation period. However, two patients who had an Irvine–Gass syndrome after cataract surgery in the first eye prior to being offered a dropless treatment regimen showed a mild pseudophakic macular oedema at the three month assessment, with less pronounced cystoid changes compared to the fellow eye at a similar postoperative time point. Both patients were asymptomatic and the macular oedema resolved using topical anti-inflammatory eye drops applied with domestic support. In hindsight, it could have been possible to repeat a subconjunctival Adcortyl injection or to observe for spontaneous resolution.

In conclusion, a dropless treatment regimen after cataract surgery is a feasible option to control postoperative inflammation in patients with difficulty using eye drops. This may be achieved with low-dose subconjunctival triamcinolone; higher dose with/without adjunctive short-acting betamethasone may be used for prophylactic control of increased inflammation, e.g. in surgically complex cases. Caution would be required in patients with glaucoma and/or a known steroid response.

## Data Availability

All data generated or analysed during this study are included in this published article. Additional specific information is available from the corresponding author on reasonable request.
